# Supplementary material for: HuR Promotes the Differentiation of Goat Skeletal Muscle Satellite Cells by Regulating Myomaker mRNA Stability
Source: Int J Mol Sci. 2023 Apr 7;24(8):6893. doi: 10.3390/ijms24086893 (PMC10138435; doi:10.3390/ijms24086893)
Supplement: Supplementary file 1 [file ijms-24-06893-s001.zip › ijms-2258904-supplementary.pdf]

Table S1. Primers used in this study.

Gene	Primer	sequence (5′-3′)	Tm/°C	Product Size/bp
CHRNA1	CHRNA1-F<br>CHRNA1-R	AGTGGGTGCGGAAGGTTTTT<br>GGGGTGTTTGATCAGGGGAG	55	182
CAPN6	CAPN6-F<br>CAPN6-R	AGCCTAGAGGACTTTTGCCG<br>TTGTGCTCATCCTCTGGCAC	57	215
MYOM3	MYOM3-F<br>MYOM3-R	GTATCAGGTCCGTCAGCGAG<br>ATATGGCGCAGACGGTGTAG	55	98
COL11A1	COL11A1-F<br>COL11A1-R	TTTTGAGGGGGACATCCAGC<br>TCAGGTGCATACTCGTCAACC	64.5	128
CDK14	CDK14-F<br>CDK14-R	TATTCCAGTTGCTGCGAGGG<br>TATGTGTGGCTAGGGACGGA	57	154
MYOZ2	MYOZ2-F<br>MYOZ2-R	ATCTCAGTAACCGTGGTGCC<br>CCGGGGTGTTAGGAGGAGTA	59	192
FMNL2	FMNL2-F<br>FMNL2-R	CAACCATACAGACGAGCCGA<br>CGGCACCATTAACAGAACGC	61.3	85
CKB	CKB-F<br>CKB-R	CCCAGGCCACCCCTATATCA<br>GCTCGTCGGTGGGCTTATAG	59	128
FGF11	FGF11-F<br>FGF11-R	ACCAGCTCTTTCACCCACTT<br>TAGAGAGCAGAGGCGTACAG	62.6	194
SYNPO2L	SYNPO2L-F<br>SYNPO2L-R	TCCAAGATCCGAAGACGGAG<br>CCCTGAGGCATCGATAAGGC	61.4	132
HuR	HuR-F<br>HuR-R	TGACCCAGGATGAGCTACGA<br>AAGCTGTGTCCTGCCACTTT	59	91
Myomaker	Myomaker-F<br>Myomaker-R	CTTCAGCATCTACGGGACGG<br>ATGGTCAGGACGCCAAACAT	55.7	108
MyHC	MyHC-F<br>MyHC-R	CCACATCTTCTCCATCTCTG<br>GGTTCCTCCTTCTTCTTCTC	60.7	177
MyoD	MyoD-F<br>MyoD-R	GTGCAAACGCAAGACGACTA<br>GCTGGTTTGGGTTGCTAGAC	60.4	128
MyoG	MyoG-F<br>MyoG-R	GGACCCTACAGATGCCCACAA<br>TTGGTATGGTTTCATCTGGG	59	101
GAPDH	GAPDH-F<br>GAPDH-R	GCAAGTTCCACGGCACAG<br>GGTTCACGCCCATCACAA	59	249
PGK1	PGK1-F<br>PGK1-R	TGGACCTGTGGGTGTATT<br>CTGACTTTATCCTCCGTGTT	59	159
Actin	Actin-F<br>Actin-R	CCTGCGGCATTCACGAAACTAC<br>ACAGCACCGTGTTGGCGTAGAG	59	87
PEGFP-HuR	PEGFP-HuR-F<br>PEGFP-HuR-R	CCGCTCGAGATGTCTAATGGTTATGAAGA<br>CCCAAGCTTTTTGTGGGACTTGTTGG	60.8	1000
